# Study protocol for a randomized controlled trial testing the efficacy of Emotion Detectives In-Out: a blended version of the unified protocol for transdiagnostic treatment of emotional disorders in Portuguese children

**DOI:** 10.1186/s40359-024-01532-z

**Published:** 2024-02-07

**Authors:** Helena Moreira, Dave Skvarc, Bárbara Gomes-Pereira, Alzira Albuquerque, Ana Carolina Góis, Ana Fonseca, Ana Maria Pereira, Brígida Caiado, Bruna Paulino, Catarina Santos, Jill Ehrenreich-May, Maria Cristina Canavarro, Mariana Saraiva, Vitória Nunes Vicente, Ana Isabel Pereira

**Affiliations:** 1 Center for Research in Neuropsychology and Cognitive-Behavioral Intervention, Faculty of Psychology and Educational Sciences, University of Coimbra, Rua Do Colégio Novo, 3030-115 Coimbra, Portugal; 2 School of Psychology, Faculty of Health, Deakin University, Geelong, VIC Australia; 3 Centro Hospitalar Tondela-Viseu, Viseu, Portugal; 4 CICPSI, Faculdade de Psicologia, Universidade de Lisboa, Lisbon, Portugal; 5 Centro Hospitalar Universitário de Lisboa Central, Lisbon, Portugal; 6 Department of Psychology, University of Miami, Miami, USA

**Keywords:** Unified protocol for children, Emotion Detectives In-Out, Coping cat, Randomized controlled trial, Children, Anxiety disorders

## Abstract

**Background:**

Childhood emotional disorders (EDs; i.e., anxiety and depressive disorders) are currently a public health concern. Their high prevalence, long-term effects, and profound influence on the lives of children and families highlight the need to identify and treat these disorders as early and effectively as possible. This clinical trial will examine the efficacy of a blended version (i.e., combining face-to-face and online sessions into one treatment protocol) of the Unified Protocol for Children (the “Emotion Detectives In–Out” program). This program is a manualized cognitive-behavioral therapy for the transdiagnostic treatment of EDs in children aged 7 to 12 years that aims to reduce the intensity and frequency of strong and aversive emotional experiences by helping children learn how to confront those emotions and respond to them in more adaptive ways.

**Methods:**

This study is designed as a multicenter equivalence randomized controlled parallel-group two-arm trial comparing the Emotion Detectives In–Out program with an evidenced-based group intervention for children with anxiety disorders (the Coping Cat program). Participants will be children aged between 7 and 12 years with an anxiety disorder or with clinically significant anxiety symptoms as well as one of their parents or a legal representative. A minimum sample size of 138 children (69 per group) is needed to test whether the efficacy of the proposed intervention is equivalent to that of the well-established Coping Cat intervention.

**Discussion:**

We expect Emotion Detectives In–Out to be a feasible and efficacious alternative intervention for treating children's EDs by allowing for a greater increase in children's access to care. A blended format is expected to overcome common barriers to treatment (e.g., parents´ lack of time to attend regular sessions) and make the intervention more accessible to families.

**Trial registration:**

The clinical trial is registered at ClinicalTrials.gov (Identifier: NCT05747131, date assigned February 28, 2023).

**Supplementary Information:**

The online version contains supplementary material available at 10.1186/s40359-024-01532-z.

Childhood emotional disorders (EDs), such as anxiety and depressive disorders, are currently considered an important public health concern [1]. The prevalence of these problems has been growing in recent years [2–4], particularly during and after the COVID-19 pandemic. A recent meta-analysis of the global prevalence of children’s and adolescents’ clinical levels of depression and anxiety symptoms during the COVID-19 pandemic estimated a pooled prevalence of 25.2% for depression symptoms and 20.5% for anxiety symptoms [5], which is more than double the estimated prevalence of both types of symptomatology in the prepandemic period. EDs in childhood significantly impact children´s functioning at multiple levels (e.g., family, academic, social) [6] and have long-term consequences. For instance, it is estimated that half of all mental health problems in adulthood have their onset before the age of 14 years [4, 6]. EDs also represent a societal burden; for example, families of children with anxiety disorders have 20 times higher costs than families from the general population [7]. It is therefore critical to recognize and treat EDs as early as possible.

Nevertheless, there is a considerable gap between children’s needs and their actual access to mental health care. The majority of children remain untreated due to a lack of access to psychological treatment, particularly evidence-based treatment (EBT) [8]. One of the key reasons for the absence of adequate mental health treatment is the inability of pediatric mental health public services to provide these children with a prompt and effective response. On the one hand, there is a lack of trained clinicians in many pediatric mental health public services. On the other hand, face-to-face therapy continues to be parents’ preferred intervention modality [9–11]. In addition, it is frequently the only delivery format offered in pediatric services, which contributes to lengthy wait lists and extended gaps between therapy sessions.

Many barriers may prevent parents from seeking face-to-face therapy for their children, such as a lack of time to attend regular sessions, costs associated with time off work and travel, and a lack of motivation to commit to lengthy face-to-face treatment [12]. Therefore, it is urgent to improve the accessibility of EBTs for childhood EDs in public mental health systems. Internet-based psychological interventions can be an effective solution for overcoming these difficulties [13, 14].

In this study, we will test the efficacy of a blended version (i.e., a combination of face-to-face and online therapy in one integrated treatment protocol [15]) of the Unified Protocol (UP) for the transdiagnostic treatment of emotional disorders in children (UP-C; [16]). The UP-C is a cognitive‒behavioral therapy (CBT) that adopts a transdiagnostic approach to treat multiple EDs simultaneously by addressing the shared mechanisms underlying these disorders (e.g., neuroticism) and combining EBT strategies (e.g., exposure, mindfulness, cognitive flexibility). It is an adaptation of the adult UP [17] for children aged 7 to 13 and delivers the key components of the UP in an interactive and child-friendly group format with considerable parent involvement. A pilot randomized controlled trial (RCT) comparing the UP-C with an anxiety-specific EBT (Cool Kids; [18]) found that both treatments were effective in treating anxiety, but the UP-C resulted in greater improvements in depression and emotion regulation [19].

In addition to its promising efficacy, the UP-C has important advantages over other EBTs, making it scalable and easily implementable in child mental health services. For instance, the UP-C is a group therapy that enables the simultaneous treatment of 7–8 children with different EDs, thereby reducing the costs associated with individual treatment for families and institutions and minimizing the therapist’s burden [20]. In addition, focusing on the core mechanisms underlying different EDs may have favorable long-term outcomes [21]. Another critical advantage is that unlike most psychological interventions for children, it includes the same number of sessions for parents that focus on parental behaviors that are known to be critical risk and maintenance factors of EDs in childhood (e.g., parental criticism, overprotection, modeling of intense emotions and inconsistency) [22]. Furthermore, there is evidence that the efficacy of CBT improves significantly when caregivers are more involved in their children’s treatment [23].

While the UP-C is a promising approach to treating childhood EDs, the demand for face-to-face therapy exceeds the capacity of child mental health services [24], and many barriers prevent parents from seeking face-to-face therapy for their children. Internet-based interventions can overcome these barriers and provide an easily accessible option that may significantly increase access to care [25]. Importantly, internet-based interventions may be particularly appealing to children, who are typically early adopters and regular users of new technologies [26]. In addition, there is evidence that internet-based interventions (e.g., BRAVE-online; [27]) are effective in reducing children’s EDs [14, 28–30] and are acceptable to children, families and clinicians [11, 31–34]. However, certain drawbacks to interventions conducted exclusively online have been recognized, including the absence of direct clinician interaction, limited effectiveness in addressing severe mental health issues, and elevated rates of participant dropout [35, 36]. A blended format can overcome these limitations and make psychological therapy more accessible to families [15].

Therefore, a blended version of the UP-C, the Emotion Detectives In–Out, was developed to improve the delivery of the UP-C by retaining the positive aspects of face-to-face and online therapy while mitigating their disadvantages [37, 38]. This new version is expected to reduce therapy costs for families and institutions, improve compliance, increase motivation, enhance the uptake of treatment principles, and facilitate the generalization of the application of core treatment components [39].

## Objectives

The main goal of this clinical trial is to test the efficacy of the Emotion Detectives In–Out intervention in reducing children’s anxiety symptoms and changing secondary outcomes in comparison to an active control group, the Coping Cat intervention, an EBT for children with anxiety [40]. A multicenter equivalence RCT will be used to answer the critical question of whether the Emotion Detectives In–Out intervention has at least as much efficacy as the active control group in reducing anxious symptomatology and changing secondary outcomes [41]. The Emotion Detectives In–Out intervention is expected to be as effective as the Coping Cat while presenting important advantages, including greater availability, reduced cost for families and institutions, a lower therapist burden, and greater parental involvement. In this clinical trial, we also aim to identify key factors that may predict adherence to the Emotion Detectives In–Out and that may predict treatment outcomes.

## Methods

### Trial design

This study is designed as an equivalence randomized controlled parallel-group two-arm trial (allocation ratio 1:1) to compare a new blended group intervention (Emotional Detectives In–Out) with a face-to-face group intervention (Coping Cat) for children with anxiety disorders. The Coping Cat intervention was chosen as the active control group because it is an evidence-based cognitive-behavioral intervention for children with anxiety disorders [40, 42] that can be used to test the equivalence of the Emotion Detectives In–Out intervention in reducing anxiety symptomatology. An equivalence trial aims to demonstrate that a novel or non-established treatment is as effective as a well-established treatment within a prespecified margin of equivalence. In the current trial, the margin of equivalence was defined as the difference between the intervention groups that would be considered the minimum clinically relevant difference.

The clinical trial is registered at ClinicalTrials.gov (identifier: NCT05747131, date assigned February 28, 2023). Authorization for sample recruitment was obtained from the Ethics Committee of the Faculty of Psychology and Education Sciences of the University of Coimbra and from the Ethics Committee of the Faculty of Psychology of the University of Lisbon. The clinical trial will follow the ethical recommendations of the American Psychological Association (APA, 2010) and the World Medical Association’s Declaration of Helsinki (World Medical Association, 2013).

The SPIRIT (Standard Protocol Items: Recommendations for Intervention Trials) checklist of information [43] and the CONSORT recommendations for noninferiority and equivalence trials [41] will be used as guidance to report the study protocol of this clinical trial.

### Participants

The participants will be children aged 7 to 12 years with an anxiety disorder or subclinical anxiety symptoms as well as one of their parents or a legal representative. The following eligibility criteria will be considered:

1. The child is aged 7 to 12 years;
2. The child has a primary diagnosis of an anxiety disorder or clinically significant anxiety symptoms;
3. Both the child and the parent/legal representative are able to speak, read and understand Portuguese;
4. Both the child and the parent/legal representative have internet access;
5. The child does not have a diagnosis of a psychotic disorder, bipolar disorder, intellectual disability or autism spectrum disorder;
6. The child does not have severe current suicidal ideation;
7. The child is on a stable dose of psychotropic medication for at least 1 month prior to the baseline assessment;
8. The child is not currently receiving other types of psychotherapy.

### Study procedures

**Fig. 1:**
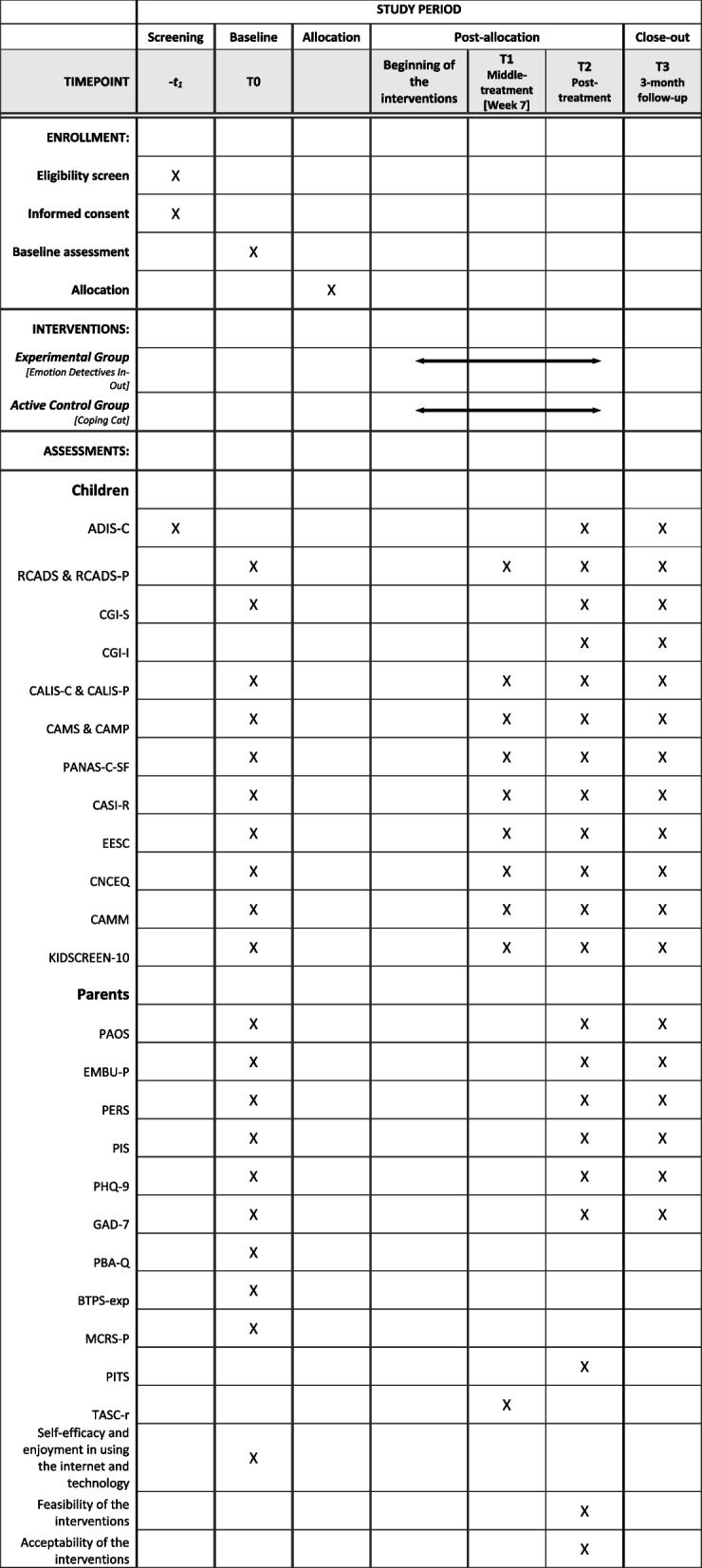
Schedule of enrollment, interventions, and assessments

The SPIRIT schedule of enrollment, interventions, and assessments is presented in Fig. 1.

#### Participant recruitment

Participants will be recruited in public hospitals and schools from different locations in Central Portugal and the Lisbon Metropolitan Area. At the child mental health services of the collaborating hospitals, children who are having a first visit with a mental health specialist will be referred to the clinical trial based on a preliminary evaluation of the child’s symptomatology and the clinical judgment that the child would benefit from a group intervention. Parents and children who agree to participate in the trial will be contacted by the research team to schedule the eligibility interview. At schools, the teachers and psychologists who are collaborating in the study will refer children previously identified as potentially having an ED. The research team will then contact the parents to confirm the main eligibility criteria and schedule the eligibility interview. Teachers will also be asked to disseminate the project via email to all parents, who will be able to enroll in the project through a link provided in the email. Finally, parents will have the opportunity to contact the research team directly via email and express their desire to enroll their child in the trial after obtaining information about the study through the project’s social media platforms or from flyers distributed at mental health services at hospitals and schools.

All parents will be informed that they are being invited to participate in a clinical trial that will assess the efficacy of two psychological interventions for children with anxiety disorders: the Emotion Detectives In–Out and the Coping Cat. They will not be informed about which intervention is the experimental or the control condition.

#### Participant selection

The eligibility criteria for the study will be assessed in an initial interview with parents and children. First, a researcher will briefly explain the goal and procedures of the study and the initial interview and will ask both the parents and the child for consent to administer the Anxiety Disorders Interview Schedule for Children (ADIS-IV-C) [44]. The ADIS-IV-C is a semistructured interview for assessing the presence of anxiety difficulties and diagnosis (e.g., separation anxiety, social phobia, specific phobia, panic disorder, agoraphobia), obsessive–compulsive disorder, posttraumatic stress disorder, depressive disorder, and externalizing disorders (e.g., attention deficit and hyperactivity disorder) in youth aged 6 to 17 years according to the diagnostic criteria of the Diagnostic and Statistical Manual of Mental Disorders (DSM-IV; APA, 1994). Parents and children will be interviewed together by a clinical psychologist, according to the procedure described by Khanna and Kendall [45]. In the case of differences between the parent's and child's responses to an item, we will adhere to the guidelines provided by Grills and Ollendick [46]. This involves consolidating both reports and employing an “OR” rule to determine the presence of a symptom or diagnosis. Therefore, information gathered from the parent and the child will be merged and considered in making the final diagnostic decisions. Each disorder will receive a clinician severity rating (CSR) ranging from 0 to 8. A CSR of 4 or greater indicates the presence of a clinical diagnosis. The principal diagnosis will be the disorder(s) with the highest CSR. If a child exhibits some symptoms of a disorder but does not meet all the criteria and these symptoms affect daily functioning, the condition will be categorized as subclinical.

The absence of a diagnosis of a psychotic disorder, bipolar disorder, intellectual disability, autism spectrum disorder, and current suicidal ideation as well whether the participants have internet access to complete online sessions and proficiency in Portuguese will also be assessed during the initial interview. The child´s medication will be confirmed with the child´s pediatrician/psychiatrist or with the parents. This initial interview is expected to last approximately 90 min.

Parents will be informed at the end of the interview if their child meets the eligibility criteria for enrollment in the clinical trial. Parents of eligible children will be provided with detailed oral and written information about the next phase of the study and will be asked to provide written informed consent, and children will be asked to provide their assent. The researchers will orally explain the main characteristics of the study and will answer all of the participants’ questions, both during the eligibility interview and throughout the implementation of the study. Parents and children will receive adequate information about the clinical trial in a way that they can understand to enable them to make an informed decision. Specifically, among other aspects that are common to the majority of clinical trials (e.g., voluntary participation in the study, guarantee of anonymity and confidentiality), the researchers will inform the parents/legal representatives about a) the experimental design of the study and its main characteristics; b) alternative treatment options if the parents and/or the child decide not to participate in the study or choose to withdraw; and c) the absence of monetary costs associated with participation. After providing informed consent/assent, the parents and children will fill out the baseline (T0) assessment measures.

If a child refuses to participate, even if the child’s parents have signed the informed consent form, the child will not be included in the study and will be referred to a mental health specialist within the health or school institution where the child was recruited. Participants who do not meet the inclusion criteria will be duly informed about the reasons for their exclusion from participation. If these reasons are due to the presence of a serious psychiatric disorder or other difficulty requiring mental health support (e.g., psychotic or behavioral problems as the main diagnosis), the parents will immediately receive recommendations about the best type of medical/psychological support, and the psychologist or psychiatrist of the health service or school will be notified.

#### Sample size

A minimum sample size of 138 children (69 per group) is needed to test the equivalence between two interventions that do not differ by more than the minimum expected effect size of *d* = 0.50, which is slightly larger than the recommended minimum practical effect size indicated by Ferguson [47]. This calculation is based on the two one-sided tests (TOST) procedure for determining equivalence as outlined by Lakens [48] and using the authors´ TOSTER package for R, assuming equal group allocation, alpha = 0.05, 80% power, and four measurements. This sample size is also sufficient to detect within-subject changes in anxiety scores of the same magnitude (*d* = 0.50) over four measurements (WebPower; [49]). With this sample and allowing for a substantial violation of sphericity, the achieved power is 97%. Finally, an *N* = 138 with four measurements is sufficient for growth mixture modeling [50]. With an attrition rate of 15% [19, 51], we will have to recruit approximately 165 parents at T0 to achieve the final intended sample size.

#### Randomization

##### Sequence generation

After the children and parents fill out the baseline (T0) assessment measures, they will be randomly assigned at each recruitment location by a researcher not collaborating in the study and through an automated, web-based randomization program to one of two conditions: the experimental condition (Emotion Detectives In–Out) and the active control condition (Coping Cat). A permuted block randomization procedure will be employed in each location to obtain groups with an equivalent number of participants. The block size will depend on the number of eligible children in each location, but it is expected to be between 10 and 12 so that 5 to 6 participants can be assigned to each condition.

##### Concealment mechanism

The allocation sequence will be generated in a web-based randomization program by a researcher who is not collaborating in the study and who has no role in determining the eligibility and entry of participants in the study. The allocation sequence will then be provided to the researchers, who will communicate the randomization result to the parents via telephone.

##### Blinding

This clinical trial will be an open-label trial because both the researchers and the participants will know which treatment they receive. However, the participants will not know which intervention is the experimental condition and which is the control condition to avoid expectations about the efficacy of the new intervention and the comparison intervention.

### Interventions

#### Emotion Detectives In-Out

The Emotion Detectives In–Out program is a blended cognitive-behavioral intervention for children aged 7 to 12 years with emotional disorders and their parents. It is a transdiagnostic and emotion-focused manualized treatment designed to help children reduce the intensity and frequency of strong and aversive emotional experiences and to help parents reduce parental emotional behaviors. The standard version [16] includes a set of CBT techniques (e.g., problem-solving, mindfulness, cognitive flexibility, exposure) that are presented to the children and parents through the analogy of an “emotion detective” and are organized into five primary sections around the acronym CLUES (C: “Consider How I Feel”; L: “Look at My Thoughts”; U: “Use Detective Thinking”; E: “Experience My Fears and Feelings”; and S: “Stay Healthy and Happy”). In the blended version, the mindfulness skill will be implemented from session 4 onward until the program's conclusion, with each session commencing with a short mindfulness exercise. The concept of self-compassion (nonjudgmental awareness) will be introduced in session 4. The blended UP-C maintains the same 15 weekly sessions for children as the standard UP-C, but 10 of these are face-to-face group sessions, three are online self-guided sessions, and two are online therapist-guided sessions through videoconference. Face-to-face sessions have an expected duration of 90 min. They will include approximately 5–6 children and will be implemented by one clinical psychologist. The online sessions are entirely self-guided and will last approximately 45 min (see Table 1).

**Table 1:** Session-by-session description of the emotion detectives in–out psychological intervention (Children)

Children		
Session	**Theme**	**Format Delivery**
1	Introduction to the Emotion Detectives In–Out program	F2F
2	Getting to know my emotions (psychoeducation about emotions)	F2F
3	My body sensations	F2F
4	Using science experiments to change our emotions and behaviors (acting opposite) and introduction to mindfulness and self-compassion	F2F
5	Identify my thoughts (flexible thinking and thinking traps)	OSG
6	Detective thinking	F2F
7	Problem-solving	OSG
8	Introduction to emotion exposure	F2F
9	Review emotion detectives’ skills and safety behaviors	OSG
10	Exposure (group exposure and individual exposures)	F2F
11	Videoconference session: Questions, feedback about the child's progress, and discussion of difficulties	OTG
12	Individual exposure	F2F
13	Videoconference session: Questions, feedback about the child's progress, and discussion of difficulties	OTG
14	Individual exposure	F2F
15	Conclusion, relapse prevention, and celebration	F2F

*F2F* Face-to-face session, *OSG* Online self-guided session, *OTG* Online therapist guided session

Parents will be asked to participate in the first and last face-to-face sessions and in five online therapist-guided sessions through videoconference and to complete 10 online self-guided sessions (see Table 2). After the parents and children complete their registration, they can access the online sessions through the program's website (https://detetivesdasemocoes.pt). The online self-guided sessions include psychoeducational videos, brief exercises, and games (e.g., matching activities, problem-solving exercises). All exercises are audio-described. Homework assignments should be completed weekly on the online platform to promote the children’s interaction with the platform and keep them motivated to complete the online sessions.

**Table 2:** Session-by-session description of the emotion detectives in–out psychological intervention (Parents)

Parents		
Session	**Theme**	**Format Delivery**
1	Introduction to the Emotion Detectives In–Out program	F2F
2	Psychoeducation about emotions and emotional parenting behaviors vs. opposite parenting behaviors. Criticism vs. positive reinforcement	OSG
3	The concept of somatization, body scanning and interceptive exposures. Criticism vs. empathy Videoconference session: Questions, feedback about the child's progress, and discussion of difficulties	OSG + OTG
4	Using science experiments to change our emotions and behaviors Criticism vs. positive reinforcement	OSG
5	The concept of cognitive flexibility and thinking traps. Different types of reinforcements and punishments. Inconsistency vs. consistent reinforcement and discipline. Nonjudgmental awareness	OSG
6	Detective thinking. Overcontrol/overprotection vs. healthy independence granting Videoconference session: Questions, feedback about the child's progress, discussion of difficulties	OSG + OTG
7	Problem-solving steps and problem-solving for interpersonal conflicts Promoting independence with detective thinking and problem solving	OSG
8	Emotion exposure vs. avoidance. Present-moment awareness vs. automatic pilot. Introduction to the Emotion Ladder Videoconference session: Questions, feedback about the child's progress, discussion of difficulties	OSG + OTG
9	Review emotion detectives’ skills and introduction to emotional exposure. Excessive modeling of intense emotions and avoidance vs. healthy emotional modeling	OSG
10	The concept of safety behaviors. How to use opposite parenting behaviors to support child’s exposure Videoconference session: Questions, feedback about the child's progress, discussion of difficulties	OSG + OTG
11	Understanding what may happen when the child starts the exposure Learning to manage challenges that often arise during exposure	OSG
12	Videoconference session: Questions, feedback about the child's progress, and discussion of difficulties	OSG + OTG
13	–	–
14	–	–
15	Conclusion, relapse prevention, and celebration	F2F

*F2F* Face-to-face session, *OSG* Online self-guided session, *OTG* Online therapist guided session

#### Coping cat

Coping Cat is a cognitive behavioral therapy for children aged 7 to 13 years with anxiety disorders. In the first segment of the intervention, the children acquire several strategies that help them manage anxiety (i.e., recognizing anxiety through the identification of body cues; managing physiological activation through relaxation and diaphragmatic breathing; identifying and modifying negative automatic thoughts through cognitive restructuring; problem-solving; self-reward). The children integrate the strategies learned into a fear plan using the acronym FEAR (F = Feeling Frightened; E = Expecting Bad Things to Happen; A = Attitudes and Actions that Can Help; and R = Results and Rewards). Once the children develop these skills, they are better able to face anxiety-producing situations. Therefore, the intervention's second segment focuses on gradual exposure to anxiety-producing situations. In- and out-session group and individual exposure tasks are conducted with the children. The group version of the Coping Cat program consists of 16 weekly group sessions with 5 to 7 children per group and two individual sessions with the parents (see Table 3). The face-to-face sessions have an expected duration of 90 min and will be implemented by one clinical psychologist.

**Table 3:** Session-by-session description of the group coping cat psychological intervention

Session	Theme	Participant
**1**	Introduction to the Coping Cat program	Child
**2**	Psychoeducation about emotions	Child
**3**	Recognizing somatic responses to anxiety	Child
**4**	Managing the somatic component of anxiety	Child
**5**	Recognizing and modifying negative cognitions	Child
**Parents’ sessions (during weeks 4 & 5)** Encouraging parental cooperation with the treatment program and gathering additional information about each child		Parents
**6**	Recognizing and modifying negative cognitions and problem-solving	Child
**7**	Developing realistic self-evaluations and using self-reward	Child
**8**	Fear plan review	Child
**Parents’ session (during weeks 8 & 9)** Updating information about each child, reviewing and summarizing the skills presented during the half part of the program, encouraging parental cooperation with the exposure segment of the treatment, and working with parents to design in vivo experiences tailored to each child		Parents
**9–11**	Exposure	Child
**12–15**	Exposure	Child
**16**	Conclusion	Child

#### Supervision and training

All therapists who facilitate the groups will have a master’s degree in clinical psychology and experience in cognitive behavioral therapy, specifically in its application to children. They will have undergone training in both programs to ensure a comprehensive understanding and proficiency in the therapeutic approaches utilized.

#### Treatment fidelity

Several procedures will be adopted to ensure that both interventions are delivered as designed. First, the Emotion Detectives In–Out and the Coping Cat therapist guides and workbooks will be provided to all therapists to ensure adherence to the intervention protocol. Second, the coordinators of the research project will provide weekly supervision to all psychologists delivering the programs. Third, after each session, each therapist and an observer (typically, a master student in clinical psychology) will complete an adherence and competence checklist and will review the responses to confirm that topics were covered as outlined in the manuals.

### Outcome measures

As depicted in Fig. 1, the parents and children will complete self-report questionnaires at baseline, mid-treatment (at 7 weeks for Emotion Detectives In–Out and 8 weeks for Coping Cat), posttreatment (at 15 weeks for Emotion Detectives In–Out and 16 weeks for Coping Cat), and follow-up (at 3 months). The (ADIS-IV-C) [44] will be administered at the screening phase, posttreatment, and follow-up.

#### Primary outcome measures

##### Anxiety symptoms

Changes in anxiety will be assessed through the ADIS-IV-C [44] as well as the Revised Children’s Anxiety and Depression Scale (RCADS) – Child and Parent Versions [52]. The RCADS assesses children’s anxiety symptoms from the perspectives of the children and parents, respectively, and includes 47 items distributed across six subscales: Separation Anxiety Disorder, Social Phobia, Generalized Anxiety Disorder, Panic Disorder, Obsessive Compulsive Disorder, and Low Mood (major depressive disorder). The scales yield an anxiety total score (the sum of the 5 anxiety subscales) and a total score (the sum of all 6 subscales). Items are rated on a 4-point Likert scale ranging from 0 (*never*) to 4 (*always*), with higher scores indicating increased symptom severity.

##### Symptom severity and improvement

The Clinician Global Impression – Severity scale [53] is a one-item measure that will be used to assess the clinician's perception of the severity of the children’s anxiety symptoms, with scores ranging from 1 (*not at all ill*) to 7 (*extremely ill*). Higher scores indicate increased symptom severity. The Clinician Global Impression – Improvement scale is also a one-item measure that will assess the clinician's perception of the children’s improvement as a result of the intervention, with scores ranging from 1 (*very much improved*) to 7 (*very much worse*). Higher scores indicate increased symptom worsening.

##### Interference of anxiety in child and family life

The Child Anxiety Life Interference Scale—Self-Report (CALIS-C) and the Child Anxiety Life Interference Scale—Parent's Report (CALIS-P) [54, 55] will be used to assess the interference of anxiety in the children’s and families’ lives. The CALIS-C consists of one 10-item scale administered to children, and the CALIS-P consists of two 9-item scales (Child Interference Subscale and Family Interference Subscale) administered to parents. Items are rated on a 5-point Likert scale (0 = *not at all* to 4 = *a great deal*). The total score of the CALIS-C ranges from 0 to 36, with higher scores indicating greater interference of anxiety symptoms in children’s lives. The total scores of the Child Interference Subscale and the Family Interference Subscale of CALIS-P range from 0 to 36, with higher scores indicating greater interference of anxiety symptoms in the lives of children and families, respectively.

#### Secondary outcome measures

##### Children’s behavioral avoidance

The Child Avoidance Measure – Self Report (CAMS) and Child Avoidance Measure – Parent-Report (CAMP; [56, 57]) are two 8-item self-report measures that assess children’s behavioral avoidance when faced with stimuli that elicit anxiety, fear, or worry from the perspectives of the child (CAMS) and the parents (CAMP), respectively. Items are rated on a 4-point Likert scale (0 = *almost never* to 3 = *almost always*). The total score ranges from 0 to 24, with higher scores indicating higher behavioral avoidance.

##### Children's positive and negative affect

The Positive and Negative Affect Schedule for Children – Short Version (PANAS-C-SF; [58]) has 10 items and is composed of two subscales: Positive Affect and Negative Affect. Items are answered on a 5-item Likert scale that ranges from 1 *(very slightly or not at all*) to 5 (*extremely*). Both subscales range from 5 to 25, with higher scores on positive affect indicating greater intensity of positive emotions and higher scores on negative affect indicating greater intensity of negative emotions.

##### Children's anxiety sensitivity

The Children's Anxiety Sensitivity Inventory-Revised (CASI-R; [59]) is a 31-item self-report scale that measures children’s anxiety sensitivity across four domains: Fear of Cardiovascular Symptoms, Fear of Publicly Observable Anxiety Reactions, Fear of Cognitive Dyscontrol and Fear of Respiratory Symptoms. In this clinical trial, a brief version of 12 items (3 items per subscale) will be employed. Items are answered on a 3-item Likert scale that ranges from 0 *(not true*) to 3 (*very true*). A total anxiety sensitivity score can be obtained by summing across all items and ranges from 0 to 36, with higher scores indicating higher anxiety sensitivity.

##### Children's difficulties in emotion expression

The Emotional Expression Scale for Children (EESC; [60, 61]) is a 16-item self-report questionnaire that assesses children’s difficulties in emotional expression (i.e., poor emotional awareness and reluctance to express emotions). Items are answered on a 5-point Likert-type scale that ranges from 1 *(not at all true*) to 5 (*extremely true*). The total score ranges from 16 to 80, with higher scores indicating greater difficulty in expressing emotions.

##### Children's negative cognitive errors

The Children's Negative Cognitive Error Questionnaire (CNCEQ; [62, 63]) is a 24-item self-report questionnaire that assesses four types of cognitive errors: Catastrophizing, Overgeneralizing, Personalizing, and Selective Abstraction. Items are answered on a 5-point scale that ranges from 1 (*nothing like I would think*) to 5 (*exactly what I would think*). A total cognitive distortion score can be obtained that ranges from 24 to 120. Higher scores indicate more distorted cognitive processes.

##### Children’s mindfulness skills

The Child and Adolescent Mindfulness Measure (CAMM; [64, 65]) is used to assess children’s mindfulness skills (i.e., children’s present-moment awareness and their nonjudgmental, nonavoidant responses to their thoughts and feelings). This questionnaire has 10 items rated on a 4-point Likert scale ranging from 0 (*never true*) to 4 (*always true*). The total score is the sum of the 10 items and ranges from 0 to 40, with higher scores indicating higher levels of mindfulness.

##### Children's quality of life

The KIDSCREEN-10 Index (parent report) assesses children’s overall levels of quality of life (physical, mental and social) as reported by their parents [66, 67]. Items are answered on a 5-point Likert scale ranging from 1 (*never/not at all*) to 5 (*always/extremely*). The sum of all items provides a global index of quality of life. The standardized scores range from 0 to 100, with higher results suggesting better quality of life.

##### Parental anxiety and overprotection

The Parental Anxiety and Overprotection Scale (PAOS; [68]) assesses Parents’ Overprotection Behaviors, Parental Anxiety and Worry, and Support of Children’s Coping Behaviors (i.e., behaviors that aim to encourage children to cope with and face situations that cause them anxiety). The scale has 20 items rated on a 5-point Likert scale ranging from 0 (*nothing*) to 4 (*a lot*). Higher levels in each subscale indicate higher levels of each parental behavior.

##### Parental criticism

Four items of the Rejection subscale of the Egna Minnen Beträffande Uppfostran Scale (EMBU-P; [69, 70]) will assess parents’ levels of criticism toward their child. Items are answered on a 4-point Likert scale that ranges from 1 (*no, never*) to 4 (*yes, always*). The total rejection score ranges from 4 to 16, with higher scores suggesting higher levels of parental rejection and criticism.

##### Parental modeling of intense emotions

The Parents' Lack of Emotional Control subscale of the Parent Emotion Regulation Scale (PERS; [71]) assesses parents’ lack of ability to modulate their own negative emotions in the presence of the child. This subscale has 5 items answered on a 5-point Likert scale that ranges from 0 (*never or almost never*) to 4 (*always or almost always*). The total score ranges from 0 to 5, with higher scores indicating a higher level of negative emotion modeling.

##### Parental inconsistency

The Parenting Inconsistency Scale (PIS; [72]) has 8 items answered on a 5-point Likert scale that ranges from 1 (*does not describe me at all*) to 5 (*describes me completely*). The total score ranges from 1 to 5, with higher scores indicating a higher level of parental inconsistency.

##### Parental depressive symptoms

The Patient Health Questionnaire-9 (PHQ-9; [73, 74]) is a brief self-report measure that will be used to monitor the severity of depression symptoms. The scale has 9 items answered on a 4-point Likert scale that ranges from 0 (*not at all*) to 3 (*nearly every day*). The total score ranges from 0 to 27, with higher scores indicating higher levels of depression severity.

##### Parental anxiety symptoms

The General Anxiety Disorder-7 (GAD-7; [75, 76]) is a brief self-report measure used to monitor the severity of anxiety symptoms. The scale has 7 items answered on a 4-point Likert scale that ranges from 0 (*not at all*) to 3 (*nearly every day*). The total score ranges from 0 to 21, with higher scores indicating higher levels of anxiety severity.

#### Other outcome measures

##### Parental beliefs about child anxiety

A brief version of the Parental Beliefs About Child Anxiety Questionnaire (PBA-Q; [23, 77]) will be used to assess parents' anxious reactions to their children's physical symptoms and parents' negative beliefs about their children's experience of anxiety. The brief version has 4 items answered on a 4-point Likert scale that ranges from 0 (*completely disagree*) to 3 (*completely agree*). The total score ranges from 0 to 12, with higher scores indicating higher levels of parents' negative beliefs about their children's experience of anxiety.

##### Parents’ perceived barriers to the intervention

The parent version of the Barriers to Treatment Participation Scale—Expectancies (BTPS-exp; [78]) assesses parents' expectations about barriers to their children's participation in treatment. The scale has 44 items answered on a 5-point Likert scale that ranges from 1 (*totally disagree)* to 4 (*totally agree*) and includes four subscales: 1) Stressors and Obstacles That Compete With Treatment; 2) Treatment Demands and Issues; 3) Perceived Irrelevance of Treatment; and 4) Problematic Relationship With the Therapist. Mean scores can be calculated for the total scale and subscales, with higher scores indicating higher levels of expected barriers.

##### Parents’ and children’s motivation to participate in the intervention

The Motivation for Change Rating Scale (MCRS) is a questionnaire developed by the research team to assess parents’ and children’s motivation to participate in the intervention. The scale has 6 items answered on a 5-point Likert scale that ranges from 0 (*nothing*) to 4 (*very much*). The total score ranges from 0 to 24, with higher scores indicating higher levels of motivation to change.

##### Parents’ and children’s involvement in therapy

The Parental Involvement in Therapy Scale (PITS; [23, 79) is composed of two sections. The first section asks the clinician to evaluate parents’ involvement in the therapy (i.e., communication with the clinician; parents’ attendance at the parents sessions; parents’ adherence to their own homework activities; and parents’ support of their children’s exposure exercises). The second section requires the clinician to assess the child's comprehension and application of the skills learned in the session (i.e., recognition and expression of emotions; somatic management skills; cognitive skills – recognizing dysfunctional thoughts and identifying alternative thoughts; and exposure and utilization of positive coping strategies) as well as the child’s adherence to the homework activities. Items are answered on a 5-point Likert scale that ranges from 0 to 4.

##### Therapeutic alliance

The revised child version of the Therapeutic Alliance Scale for Children (TASC-r; [80]) will be administered at sessions 6 and 12 of each program to evaluate the therapeutic alliance from the perspective of the child. It includes 12 items rated on a 4-point Likert scale ranging from 1 (*not at all*) to 4 (*very much*). The items assess the child’s affect toward the therapist (e.g., “I liked spending time with my therapist”) and collaborative aspects of the therapeutic relation (e.g., “I work with my therapist on solving my problems”). The total score is computed through the sum of the 12 items.

##### Parents' and children's self-efficacy and enjoyment in using the internet and technology

Parents' and children’s self-efficacy and enjoyment in using technology will be measured by a questionnaire developed by the research team. Each scale has 3 items answered on a 5-point Likert scale that ranges from 0 (*nothing*) to 4 (*very much*). The total score ranges from 0 to 12, with higher scores indicating higher levels of self-efficacy and enjoyment.

##### Feasibility of the intervention

The feasibility of the programs will be measured through adherence (number of sessions attended and number of treatment completers, defined as those who participated in at least 70% of the sessions) and dropout rates (number of participants who dropped out from the intervention before completing it). In the Emotion Detectives In–Out condition, the pattern of online session usage by both children and parents will be assessed based on the number of completed online sessions, the timing of the completed online sessions (within the designated week or not), the number of pages accessed in each session, the number of website logins, the number of interactive exercises completed in each session, and the completion of home assignment tasks within the program platform.

##### Acceptability of the intervention

The acceptability of the interventions will be measured through specific questions developed by the research team to assess the participants’ experiences and perceptions of the intervention after each session and at the end of the program. The Child's Weekly Session Assessment Form includes eight questions (after face-to-face sessions of both conditions) or 11 questions (after online sessions in the Emotion Detectives In–Out) that assess aspects such as the child’s opinion of the level of enjoyment of the session and the games/exercises, his or her understanding of the session content, the appropriateness of the session duration, and the usefulness of homework assignments. The weekly session assessment form after the online sessions also includes three open-ended questions (“What did you like the most?”; “What did you like the least?”; “Would you change anything in the session?”). The Parents’ Weekly Assessment Form in the Emotion Detectives In–Out condition comprises nine questions that evaluate aspects such as their assessment of the usefulness of the session content and supplementary materials, their degree of satisfaction with the session, and their evaluation of the usefulness of homework assignments. Parents in the Coping Cat condition will respond to only two questions related to the support they provided to their children in completing their homework.

At the end of the program, parents and children in both conditions will complete a Final Evaluation Form that assesses several aspects, including their overall level of satisfaction with the program, their perception of the psychologist’s competence, the usefulness of the skills acquired, their intent to continue using these skills, the utility of the online platform, the user-friendliness of the online platform, and their intention to recommend or utilize the program in the future if necessary.

### Data management

The therapists who will facilitate the groups and research assistants of the research project will be responsible for administering the paper questionnaires and entering the data electronically on a SPSS database. This database will be shared exclusively among the research teams at the University of Coimbra and the University of Lisbon, with access limited to the therapists, research assistants, and project coordinators. To ensure security, all electronic files will be password-protected, and physical copies of the questionnaires will be will be safely stored in locked file storage at each university. The data and research documents will be preserved for a period of 10 years following the conclusion of the study.

### Statistical methods

#### Efficacy evaluation

The efficacy of the Emotion Detectives In–Out in comparison with the Coping Cat will be assessed by analyzing the changes in the primary and secondary outcomes. As an equivalence clinical trial, the aim is to test whether the Emotion Detectives In–Out (experimental condition) is as effective as the Coping Cat (control group). The planned analysis for each outcome is a repeated-measures linear mixed model adjusted for covariates. The primary equivalence testing will be the between-group difference TOST at postintervention and at 3 months using the Welch correction for heterogeneous variance if needed. We will also investigate the linear and nonlinear (quadratic) effects for time and condition and simple effects for parameter estimates within each condition. For categorical variables (e.g., treatment response as assessed by CGI-I), treatment conditions will be compared using Pearson’s chi-square test.

To examine for heterogeneous intervention response, we will calculate Reliable Change Indices (RCI) for all participants at postintervention and at the 3-month follow-up. The RCI will be calculated for all outcomes and correlated with demographics to examine for subgroup efficacy predictors. The cutoff for clinically significant change is generally accepted as ± 1.96 [81]. Likewise, we aim to test the longitudinal trajectory of anxious symptoms over time using Growth Mixture Modeling (GMM). Furthermore, assuming that heterogeneous trajectories of anxious symptoms are observed, we aim to test the suitability of the collected demographic variables as predictors of group membership.

For the primary equivalence, efficacy, RCI, and chi-square analyses, we will use Jamovi software and the GAMLj and TOSTER packages as needed [82]. For GMM analyses, we will use R software with the package *lccm* [83] to perform growth mixture modeling, and evaluate various model specifications of *k* classes two through six, and using both fixed and random effects. We will select the optimal number of clusters was based on four criteria: parsimony, entropy approaching 1, lowest BIC/SABIC, and minimum class size > 10% of the sample [84, 85].

Following Kennedy, Bilek [19], the attainment of diagnostic remission for both the principal diagnosis and all emotional disorder diagnoses at posttreatment and follow-up will be defined as the absence of the principal diagnosis and the absence of all emotional disorder diagnoses, respectively (i.e., ADIS-IV CSR < 4). A CGI-I score of 1 (*very much improved*) or 2 (*much improved*) at posttreatment or follow-up will be considered an indication of a meaningful reduction in the severity of emotional disorders and will be deemed indicative of a positive treatment response, consistent with the findings from other studies [19, 86].

#### Planned and post hoc examination of potential confounds and moderator effects

##### Covariates

Variables that are expected to correlate with but not predict outcomes differentially will be included in the baseline model to account for their effect. The targeted covariates include sociodemographic and clinical variables, such as the children’s age and sex, number of comorbid disorders, use of psychopharmacological medication, and previous psychological treatment.

##### Planned moderation analysis

Some variables will be analyzed as moderators of the efficacy of the Emotion Detectives In–Out in the primary and secondary outcomes, including the parents’ and children’s motivation to participate in the intervention, the frequency of homework assignments, both parents’ and children’s involvement in therapy, parents’ perceived barriers to the intervention, and both parents' and children's self-efficacy and enjoyment in using the internet and technology.

#### Attrition, nonadherence and missing data

All analyses will be performed as an intention-to-treat (ITT) analysis to ensure that the clinical efficacy of the treatment is not overestimated as an artifact of attrition (i.e., including all parents/children who completed the baseline). Missing data will be reported, and when MCAR (missing completely at random) or MAR (missing at random) occurs, multiple imputation methods will be used to estimate missing values when appropriate.

### Data monitoring

No data monitoring committee, interim analyses, or auditing will be implemented for the study. The research team will engage in ongoing discussions about important ethical issues, particularly those associated with adverse events or unintended effects, throughout the trial. For example, in cases where a serious psychological problem (such as psychotic symptoms or suicidal ideation) is identified during the eligibility interview and cannot be addressed within the current study, immediate communication will be established with the parents/legal representatives of the children. In such instances, the child will be promptly referred to a pediatric mental health service. Furthermore, if a child experiences a worsening of symptoms between the enrollment phase and the start of the intervention, their parents/legal representatives will be promptly notified. The research team will conduct a thorough assessment, and the need for referral to another type of intervention will be considered.

### Ethics and dissemination

Authorization for sample recruitment was obtained from the Ethics Committee of the Faculty of Psychology and Education Sciences of the University of Coimbra and from the Ethics Committee of the Faculty of Psychology of the University of Lisbon. Parents/legal representatives of eligible children will be requested to provide written informed consent, while the children themselves will be asked to provide their assent. All parents/legal representatives will receive information about the voluntary nature of their participation and that of their children. Those recruited at the child mental health services of the collaborating hospitals will be informed that declining to participate in the study will not impact the medical/psychological care available to their children. Additionally, parents/legal representatives will be informed that they have the right to drop out from the study at any time and for any reason (or without specifying a reason), without influencing the care provided. In cases where children or parents express a preference for individual psychotherapy rather than on the offered group interventions, they will be directed to a child psychologist for individual CBT, and their case will be excluded from the trial. All parents/legal representatives will be informed that all data collected will be utilized only at an aggregate level (e.g., average scores of all participants in the questionnaires) and that participants' identities will not be revealed in any reports or publications resulting from the study. After study completion, potential identifiers, such as study ID, will be deleted from the database (database anonymization).

The results of the study will be disseminated to both the scientific and general communities through various means. Dissemination outputs will include fact sheets, research conference posters and oral presentations, as well as scientific articles submitted to international peer-reviewed journals. The authors of these outputs will be members of the research team who have made significant and direct contributions to the study design, data collection and analysis, and manuscript writing. The research team also aims to disseminate the study findings to professionals in the field of child mental health, such as pediatricians, child psychologists or psychiatrists, school teachers, and psychologists. This dissemination will be achieved through lectures, information sessions, and/or the distribution of informative brochures at healthcare centers, hospitals, or schools.

## Discussion

The goal of this clinical trial is to compare the efficacy of a new delivery format of an effective psychological intervention for children’s EDs (the UP-C; [87]) in comparison with an established EBT (the Coping Cat). We anticipate that the Emotion Detectives In–Out will be a viable and cost-effective alternative intervention for treating children's EDs that allows for a large increase in children's access to care (e.g., fewer clinic visits, lower costs). We also expect it to increase children’s adherence, decrease dropout, and improve transfer to everyday life (e.g., online sessions support behavior change during face-to-face sessions and will be available when convenient), which will contribute to the intervention's efficacy. This new intervention may also result in significant cost savings for health institutions (due to fewer clinical consultations) and more time available for therapists, which in turn may contribute to the reduction of waiting lists and the timely attendance of children in need. In addition, by targeting common transdiagnostic mechanisms across several disorders, the Emotion Detectives In–Out intervention may eliminate the burden of training for several single-disorder protocols as therapists only need to learn this intervention to provide evidence-based care for the most common childhood EDs.

### Supplementary Information

**Additional file 1.**

## Data Availability

No datasets were generated or analysed during the current study.
